# Heredity and Environment

**Published:** 1915-09

**Authors:** Edwin G. Franklin


### Heredity and Environment.

Never before has this long discussed question assumed such relative importance as it has at the present time. This is because of the great advancement made in the study of heredity. These new discoveries have given a different view of the nature of man and has in no small way influenced philosophical thought. Of the recent books written on the subject, this one,* by a Princeton authority, is altogether the most lucid and entertaining the writer has ever read. A careful review of it will renew one's interest in the subject of genetics, and enlighten the philosophic view of life. We could give the readers of this department no more profitable matter than a review of the book.

"One of the greatest results of the doctrine of organic evolution," the author says, "has been the determination of man's place in nature, * * * that he is a part of the great world of living things, and not a being who stands apart in solitary grandeur in some isolated sphere. * * * All the general laws of life which apply to animals and plants apply also to man. * * * If 'the proper study of mankind is man,' the proper study of man is the lower organism in which life processes are reduced to their simplest terms, and where, alone, they may be subjected to conditions or rigid experimentations. * * * If human evolution may be controlled to even a slight extent, we may expect that sooner or later the human race will be changed for the better. * * * We hear much nowadays about man's control over nature, though in no single instance has man ever changed any

---

**Heredity and Environment in the Development of Men.* By Edwin Grant Franklin, Princeton University Press, Princeton, 1915.

law or principle of nature. What man can do is to put himself into such relations to natural phenomena that he may profit by them, and all that can be done toward the improvement of the human race is to apply consciously to man those great principles of development and evolution which has been operating unknown to man through all the ages."

From this declaration of facts, the author proceeds to elucidate the factors of development, explaining very clearly the character of the cell and its fertilization. As the purpose of the book is to point out the relative significance of all the factors in development, due consideration is given to the mind. "Whatever the ultimate relation of the mind and body may be, there can be no reasonable doubt that the two develop together from the germ. * * * The mind is related to the body as function is to structure, * * * the body or brain is not the cause of mind, nor mind the cause of body or brain, but that both are inherent in one common organization or individuality." Thus the development of the reflexes, instincts, intellect and will are accounted for on the same basis as the development of physical characters.

"The development of all of these psychical faculties runs parallel with the development of bodily structures, and apparently the method of development in the two cases are similar, viz., progressive differentiation of complex and specialized structures and functions from relatively simple and generalized beginnings, * * * there are in germ cells many different structures and functions which are, however, very unlike those of the adult, and by the transformation and differentiation of this germinal organization the complicated organization of the adult arises. Development is not the unfolding of an unfolded organism, nor the mere sorting of materials already present in the germ cells, though this does take place, but rather it consists in the formation of new materials and qualities, or new structures and functions—by the combination and interaction of the germinal elements present in the oosperm. * * * Unquestionably, the factors or causes of development are to be found not merely in the germ, but also in the environment; not only in intrinsic but also in extrinsic forces; but it is equally certain that the directing and guiding factors of development are in the main intrinsic, and are present in the organization of the germ cells, while the environmental factors exercise chiefly a stimulating, inhibiting or modifying influence on development."

In explaining the cellular bases, the author summarizes his discussion by saying: "The cell is the ultimate independent unit of organic structure and function. The only living bond between one generation and the next is found in the sex cells, and all inheritance must take place through these cells. Inherited traits are not transmitted from parent to offspring, but the germinal factors or causes are transmitted, and under proper conditions of environment these give use to developed characters. Every oosperm as well as every developed organism differs more or less from every other one, and this remarkable condition is brought about by extremely numerous permutations in the distribution of certain parts of the sex cells in maturation and fertilization."

In speaking of the phenomena of inheritance, the author states that children are never exactly like their parents, but are somewhat like them, i. e., there are general resemblances but particular differences between parents and their offspring. The offspring inherits certain racial characteristics and general resemblances, also certain pathological and physiological peculiarities as well as psychological characters, e. g., brachydactylism, longevity, obesity, particular instincts, aptitudes, genius, weak-mindedness, etc.

On the question of inheritance of disease, the author states: "If a disease is due to some defect in the hereditary constitution, it is inherited; otherwise, according to our definition of heredity, it is not. Of course, no disease develops without extrinsic causes, but when one individual takes a disease while another, under the same conditions does not, the differential cause may be an inherited one, or it may be due to differences in the previous conditions of life. There is no doubt that certain diseases run in families and have the appearance of being inherited, but in this case, as in many others, it is extremely difficult in the absence of experiments to distinguish between effects due to intrinsic causes and those due to extrinsic ones."

Mendel's theory is clearly explained as follows: "(a) *The Principle of Unit Characters.*—The total heritage of an organism may be analyzed into a number of characters which are inherited as a whole and are not further divisible; these are the so-called 'unit characters' (de Vries). (b) *The Principle of Dominance.*—When contrasting unit characters are present in the parents, they do not, as a rule, blend in the offspring, but one is dominant and usually appears fully developed, while the other is recessive, and temporarily drops out of sight. (c) *The Prin-*

*ciple of Segregation.*—Every individual germ cell is 'pure' with respect to any given unit character, even though it comes from an 'impure' or hybrid parent. In the germ cells of hybrids, there is a separation of the determiners of contrasting characters so that different kinds of germ cells are produced, each of which is pure with regard to any given unit character." * * *

The author makes it very plain that it is not the character itself that is inherited but the determiner in the organization of the fertilized cell that is responsible for development. Environment or extrinsic stimuli, however, determines the cause of the development. That is to say, the development of the organism will be pretty much what the environment can make it, but always within the limitation of the inherited possibility. While certain stimuli may affect the cell before and after fertilization and change the result of its development, such changes are not inherited characters. Thus the author speaks: "That unusual conditions of food, temperature, moisture, etc., may affect the germs of future generations so as to produce general indefinite variations is very probable, but this is a very different thing from the inheritance of acquired characters. The germ cells being a part of the parental organism may be modified by such changes in the environment as affect the body as a whole, they may be well nourished or starved, they may be modified by changed conditions of gravity, salinity, pressure, temperature, etc., and these modifications of the germ cells may and probably do lead to certain general modifications of the adult, which may be larger or smaller, stronger or weaker, according as the germ is well or poorly nourished, etc. It is further possible that these changed environmental conditions may bring about such changes in the structure of the germ cell as to produce great and remarkable peculiarities in the adult; but it is inconceivable that the environment that produces rickets, or hypertrophied heart, or loss of sight in one generation, should modify the germ cells of the next generation in such a peculiar and definite way that they should give rise to these particular peculiarities, in the absence of extrinsic causes which first produced them. The inheritance of acquired characters is inconceivable, because the egg is a cell and not an adult organism; and in this case there is no sufficient evidence that the thing which is inconceivable really does happen."

He further says: "Only that environment and training is good which leads to the development of good habits and traits and to the suppression of bad ones. What we commonly call 'good environment' is frequently the worse possible; what is often called a bad environment may be the best possible. We are all strangely blind with regard to these matters."

Because of our social inheritance, he points out "that the extrinsic conditions of life continue to grow more complex age after age, while our inherited natures remain unchanged."

Regarding eugenics, the betterment of the human race by eugenic methods, the author is not so encouraging, though his explanations are very convincing: He says: "All that man now is he has come to be without conscious human guidance. If evolution has progressed from the ameba to man without human interference, if the great progress from ape-like men to the most highly civilized races has taken place without conscious human control, the question may well be asked, Is it possible to improve natural method of evolution? It may not be possible to improve the method of evolution, and yet by intelligent action it may be possible to facilitate that method. Man cannot change a single law of nature, but he can put himself into such relations to natural laws that he can profit by them."

Finally, philosophizing on the principles enunciated, the author observes that "modern studies of development are profoundly changing the opinions of men with respect to human personality. Observation of the relentless laws of heredity, of the inevitable influences for good or bad of environmental conditions over which the individual has no control, undoubtedly tends to produce a sense of helplessness and hopelessness. What light is thrown upon the great problems of freedom and determinism, of responsibility and irresponsibility, of duty and necessity by modern studies of development? Such questions cannot be dealt with quantitatively and experimentally, and they lie outside the field of exact science, but they are involved in all inquiries which have to do with rational and social beings; they lie at the foundation of the application of science to human welfare; they occupy a large place in the thought and conduct of all men."

This short review is but an indication of the scope of the book but should serve to show the practical knowledge and richness of conception which it contains throughout each chapter.

---

Dr. Frank Long has decided to give up his place at the St. Joseph Hospital, Memphis, Tenn., and has associated himself with his father in the practice of medicine and surgery in Sulphur Springs.

